# Book Reviews

**Published:** 1799-06

**Authors:** 


THE VETERINARY ART.

*Memoria sull actuale epidemia de Gatti.*—Memoir on the Epidemic raging among Cats, by Valeriano Luigi Brera, Professor of Medicine, 4to. 26 pp. *Pavia.*

This ingenious memoir on the diseases of a very useful domestic animal, is addressed to the central administration of the department of Pisa; and contains contains a feries of obfervations well deferving the attention of thofe who wifh to inveftigate, with precifion, the epidemics which have often been noticed among them, in this country, particularly during the laft year.

The author gives the following accurate defcription of the malady, as obferved by him, at Pavia:

"The cat when firft attacked becomes weak, heavy, and unquiet; it avoids the fight of its mafter, conceals itfelf, refufes to eat or to drink, and is no longer attracted by the fmell of valerian, marjorum, &c. of which it is generally fo exceffively fond. As the malady increafes, the cat with difficulty fupports itfelf on its paws; its hair becomes erect, its tail droops, its eyes lofe their luftre, and the abdomen becomes difordered, it makes vain efforts to vomit, and at length finks under the difeafe.

From thefe fymptoms Brera concludes that the malady is a nervous fever, which although dangerous, is neverthelefs curable. Diffection afforded us no morbid appearances, but fome lived fpots on the bladder, and the gall-bladder dilated and filled with very black bile, the ordinary effect of nervous complaints; nor could he find any trace of worms, from which he concludes, that the difeafe is derived from the air. Valerian, marjoram, &c. diffolved in wine, and alum, garlick, &c. in fpirits, were found extremely beneficial in the cure of the difeafe. As preventive means he recommends "the killing of the animals infected, and burying them in deep pits; the purification of the air in the apartments where any have died; the adminiftration of a more than ordinary quantity of nutritive food, and the expofing them to fumigations of vinegar."

The work upon the whole contains much valuable information; and the purring tribe, which has already found a Homer in Mr. Defherbier, has now an Efculapius in Dr. Brera.

*Annals of Medicine, for the Year* 1798, *exhibiting a concife View of the lateft and moft important Difcoveries in Medicine and Medical Philofophy.* By Andrew Duncan, Sen. M. D. and Andrew Duncan, Jun. M. D. Fellows of the Royal College of Phyficians, Edinburgh. Vol. iii. 556 pp. 1799. (Price 7s. boards) *Edinburgh*, Mudie; *London*, Robinfons; and *Hamburgh*, Perthes.

Tha learned authors of thefe 'Annals' have, as ufual, divided the work into four diftinct fections. In the former of thefe they give an 'Analyfis' of twenty-three medical books, among which, the firft by Dr. *Currie*, on the effects of water as a remedy in fever; the fixth by *Von Humbolt*, on ftimulated mufcular and nervous fibres; the ninth by *Profeffor Soemmering*, on the organ of the foul; and the nineteenth by Mr. *Thomas Brown*, (a promifing young philofopher) on the Zoonomia, appear to us the moft confpicuous and interefting.

The fecond fection of the volume before us is intitled, '*Medical Obfervations*', and contains an account of nine different publications on various medical and chirurgical cafes, each of which deferves a place in this collection.

The third fection is devoted to 'Medical News', from which we have already extracted an article of a practical tendency, in our fecond Number, p. 184.

The fourth fection furnifhes us with a 'Lift of New Books' which have appeared during the year 1798, on fubjects of medicine, furgery, natural hiftory, and other branches of medical fcience.

*Briefe eines Arztes,* &c.—*Letters of a Phyſician, on ſubjects of Medicine and Politics.* By G. Wardenburg. Vol. 1. Part 1. 8vo. 270 pp. 1798. *Goettingen,* Schroeder.

These 'Letters' contain but a ſmall portion of important political matter, but they abound in medical and chirurgical remarks of an uſeful tendency. During his occaſional reſidence at Paris, or while travelling with the French armies, from May, 1796, to September 1797, the author has collected a number of valuable facts relative to the ſtate of the healing art, as well as the medical juriſprudence of France. His obſervations, however, would have been better entitled to praiſe, had they been arranged on a more ſcientific plan. Neverthelefs, conſidered upon the whole, his contributions form a valuable acquiſition to the modern hiſtory of Medicine.

The author, in conformity with other intelligent medical travellers, conſiders the prevailing diſtinction between the practice of medicine and ſurgery as one of the greateſt obſtacles to the progreſs of the healing art; a diſtinction remarkably prevalent, and more injurious, in France, than in any other country *.

*Travels in England, Scotland, and the Hebrides; undertaken for the purpoſe of examining the ſtate of the Arts, the Sciences, Natural Hiſtory and Manners, in Great Britain: Containing Mineralogical Deſcriptions of the country round New Caſtle; of the Mountains of Derbyſhire; of the Environs of Edinburgh, Glaſgow, Perth, and St. Andrews; of Inverary and other parts of Argyleſhire; and of the Cave of Fingal.* In two Volumes with plates. Tranſlated from the French of B. Faujas Saint Fond, Member of the National Inſtitute, and Profeſſor of Geology, in the Muſeum of Natural Hiſtory at Paris. 8vo. 361 and 352 pages (Price Fourteen Shillings, Boards) *London*: Ridgway.

Theſe intereſting 'Travels' contain a large fund of uſeful and truly practical information, particularly on ſubjects of natural hiſtory, and the ſtate of the arts in this country. What renders this work ſtill more valuable to the Engliſh reader, is the animated and apparently impartial ſketches of many eminent characters, to whom our learned traveller had the good fortune to be introduced, and of whom he generally ſpeaks in grateful and handſome terms. Of this deſcription are, Sir Joſeph Banks, Dr. Whitehurſt, M. Cavallo, Dr. Lettſom, J. Sheldon, W. Herſchel, Sir Henry Inglefield, Dr. Swediauer, Mr. M'Donald, of Sky, Mr. M'Lean, of Torloiſk, Meſſrs. M'Comie, and M'Gregor, of Perth, the late Principal, Dr. Robertſon, Dr. Adam Smith, Dr. Black, Dr. Cullen, Dr. Henry, and ſeveral other gentlemen of Mancheſter, Dr. Pearſon, Dr. Withering, Mr. Benjamin Watt, and Dr. Prieſtley.

As we propoſe to extract from theſe Travels, which abound in uſeful remarks, whatever has a *practical tendency*, in future numbers of our Journal, under the head of 'Medical and Phyſical Intelligence,' we ſhall at preſent only obſerve, that we have ſeldom peruſed a ſimilar work with equal ſatiſfaction, or left it with ſimilar regret.

*Précis d'Obſervations,* &c.—*A Summary Account of the principal Hot Mineral Springs of the Higher Pyrennees, particularly thoſe of St. Saviour, with Caſes of Cures.* By Cit. Fabas. 8vo. *Paris.*

The author begins with a deſcription of the Mineral Springs of St. Saviour, one of which he ſtates to be of the temperature of 29° of Réaumur's thermometer. He next proceeds to inquire into the cauſes of the heat of ſuch ſprings,

* Vide our firſt Number, p. 81. and foll.

ſprings, and enumerates the principal medicinal properties of the mineral waters of the Pyrennees, and the volatile ſubſtances they contain, particularly thoſe of St. Saviour. Theſe laſt, as the author ſhews from a number of caſes, act as *vulnerary detergents*, antiſpaſmodics, lithontriptics, depuratives, &c.

*Explication des mots d'uſage en Anatomie et Chirurgie, &c.—An Explanation of the terms uſed in Anatomy and Surgery: with a general table of diſeaſes, of operations, inſtruments, and medicines.* By Cit. Allouel. 12mo. 360 pp. (2 livres, 75 cent.) *Paris.* Remont.

We find in this work a new plan reſpecting the articulations of the bones; ſome remarks on different points relative to ligaments and cartilages; and reflections on the luxation of the humerus, and the rupture of the *burſæ mucoſæ.*

*Redenvoering, &c.—A Diſcourſe delivered at the opening of the Society of Medicine and Surgery, lately eſtabliſhed at Antwerp.* By Pierre Etienne Kok, m.d. 8vo. 51 pp. *Antwerp.* Schaefeites.

The motto of this infant ſociety is, '*Occidit qui non ſervat;*' perhaps the converſe of the propoſition would be fully as applicable—'*Servat qui non occidit.*' Be this as it may, Dr. Kok has here publiſhed an excellent inaugural diſcourſe, which contains, 1. a ſuccinct Hiſtory of Medical Science; 2. an Inquiry into the duties of a phyſician; 3. the practical deſign of the ſociety's motto; and 4. an earneſt exhortation to every citizen, to concur in promoting the ſalutary and benevolent purpoſes of a ſociety, inſtituted for the advancement of medicine, ſurgery, and the obſtetric art.

---

